# Opportunities for strengthening CTSA evaluation — CORRIGENDUM

**DOI:** 10.1017/cts.2019.429

**Published:** 2020-01-20

**Authors:** Tanha Patel, Julie Rainwater, William M. Trochim, Julie T. Elworth, Linda Scholl, Gaurav Dave, the Members of the CTSA Evaluation Guidelines Working Group

In the above article by Patel et al. [1], the affiliation of author William M. Trochim was incorrect. The correct affiliation is “Clinical and Translational Science Center, Weill Cornell Medicine, New York, NY, USA”.

The original article has been corrected online to rectify this error.

The authors regret this error.
